# Molecular Analysis of Human Respiratory Syncytial Virus Group B Strains Isolated in Kenya Before and During the Emergence of Pandemic Influenza A/H1N1

**DOI:** 10.1111/irv.70082

**Published:** 2025-02-20

**Authors:** Julia Wangui, George Gachara, Victor Mobegi, Charles Agoti, James Otieno, Silvanos Opanda, Benjamin Opot, Joseph N. Ngeranwa, Regina Njeru, Wallace Bulimo

**Affiliations:** ^1^ Centre For Virus Research Kenya Medical Research Institute (KEMRI) Nairobi Kenya; ^2^ Department of Biochemistry Kenyatta University Nairobi Kenya; ^3^ Department of Medical Laboratory Sciences Kenyatta University Nairobi Kenya; ^4^ Department of Biochemistry University of Nairobi Nairobi Kenya; ^5^ Department of Epidemiology and Demography Kenya Medical Research Institute (KEMRI) ‐ Wellcome Trust Program Nairobi Kenya; ^6^ Theiagen Genomics Highlands Ranch Colorado USA; ^7^ International Livestock Research Institute Nairobi Kenya

**Keywords:** COVID‐19 pandemic, evolution, genetic variability, HRSV‐B genotypes, pandemic influenza, recombination, SARS‐CoV‐2, spatial–temporal trends

## Abstract

**Background:**

We conducted a retrospective study to explore molecular insights into human respiratory syncytial virus (HRSV) group B strains among patients attending outpatient clinics at government medical facilities both prior and during the onset of Influenza A/H1N1/2009 pandemic outbreak.

**Methods:**

We screened 2300 nasopharyngeal swabs using multiplex real time reverse transcriptase polymerase chain reaction. We amplified a segment of the first and second hypervariable regions, as well as the conserved portion of the third domain of the G‐gene using HRSV‐B specific primers, sequenced by Sanger di‐deoxy chain termination method and thereafter analyzed the sequences.

**Results:**

We characterized the circulating strains into three known genotypes: SAB4 (1.4%), BA7 (1.4%), and multiple variants of BA9 (97.2%). The majority of BA9 viruses were uniquely Kenyan with only 4% aligning with BA9 lineages found elsewhere. The mean evolutionary rate of the HRSV‐B was estimated to be 3.08 × 10^−3^ substitutions per site per year.

**Conclusion:**

Our findings indicate that the circulating HRSV‐B viruses in Kenya underwent a slower evolution during the period of 2007–2010. Additionally, our findings reveal the existence of a unique lineage as well as new variants that have not been reported elsewhere to date.

## 1 Introduction

Human respiratory syncytial virus (HRSV) is a leading viral pathogen that causes acute lower respiratory tract infections (ALRI) in children resulting in hospitalization and severe illness in the elderly and immuno‐compromised populations globally [1, 2]. The virus is estimated to cause over 30,000 million ALRI in children under the age of 5, resulting in up to 199,000 deaths annually [3].

HRSV is enveloped with a negative‐sense, single‐stranded RNA genome of approximately 15.2 kb. It belongs to *Orthopneumovirus* genus of the *Pneumoviridae* family [4, 5]. It is classified into two HRSV groups (A and B) and further categorized into various genotypes [6, 7]. The nucleotide sequence variability in the C‐terminus of the G protein has been widely utilized to classify HRSV into genotypes until recently with efforts to standardize the classification HRSV genotypes [8, 9]. The allocation of genotypes in HRSV has been a topic of ongoing debate within the scientific community. Despite the absence of a unanimous consensus regarding the criteria for genotype classification, a myriad of HRSV‐B genotypes persistently emerges in research findings. Notable among these are GB 1‐5, SAB 1‐4, BA1‐14, BA‐C, BA‐CCA, BA‐CCB, CB1, JAB1, NZB1, and NZB2 [9, 10, 11, 12]. A new proposed classification system reduces the number of genotypes into seven (GB1–GB7) [9].

HRSV‐B with a 60‐nucleotide (20‐amino acid) duplication was first reported in Buenos Aires, Argentina, in 1999 [13]. As the decade drew to a close, a noteworthy phenomenon in the virological landscape unfolded with the global dissemination of genotypes in this subgroup (BA subgroup). It was first detected in Kenya in 2003, and within a decade, it had replaced the existing genotypes in the country with exception of the sporadic SAB4 also reported elsewhere [14, 15, 16]. Over the past decade, the BA9 genotype has exhibited a growing prevalence, gradually asserting its dominance on a global scale and consequently displacing other genotypes [17, 18, 19].

The primary objective of this study was to genetically profile and deduce the evolutionary dynamics of human respiratory syncytial virus subgroup B (HRSV‐B) among individuals exhibiting influenza‐like illness (ILI). These were a proportion of patients seeking treatment at eight public hospitals in the country during the period from 2007 to 2010. It is noteworthy that this timeframe corresponds to the time just before and during the emergence of the influenza pandemic attributed to the novel A/H1N1 pandemic strain.

## 2 Materials and Methods

### 2.1 Study Population

**FIGURE 1:**
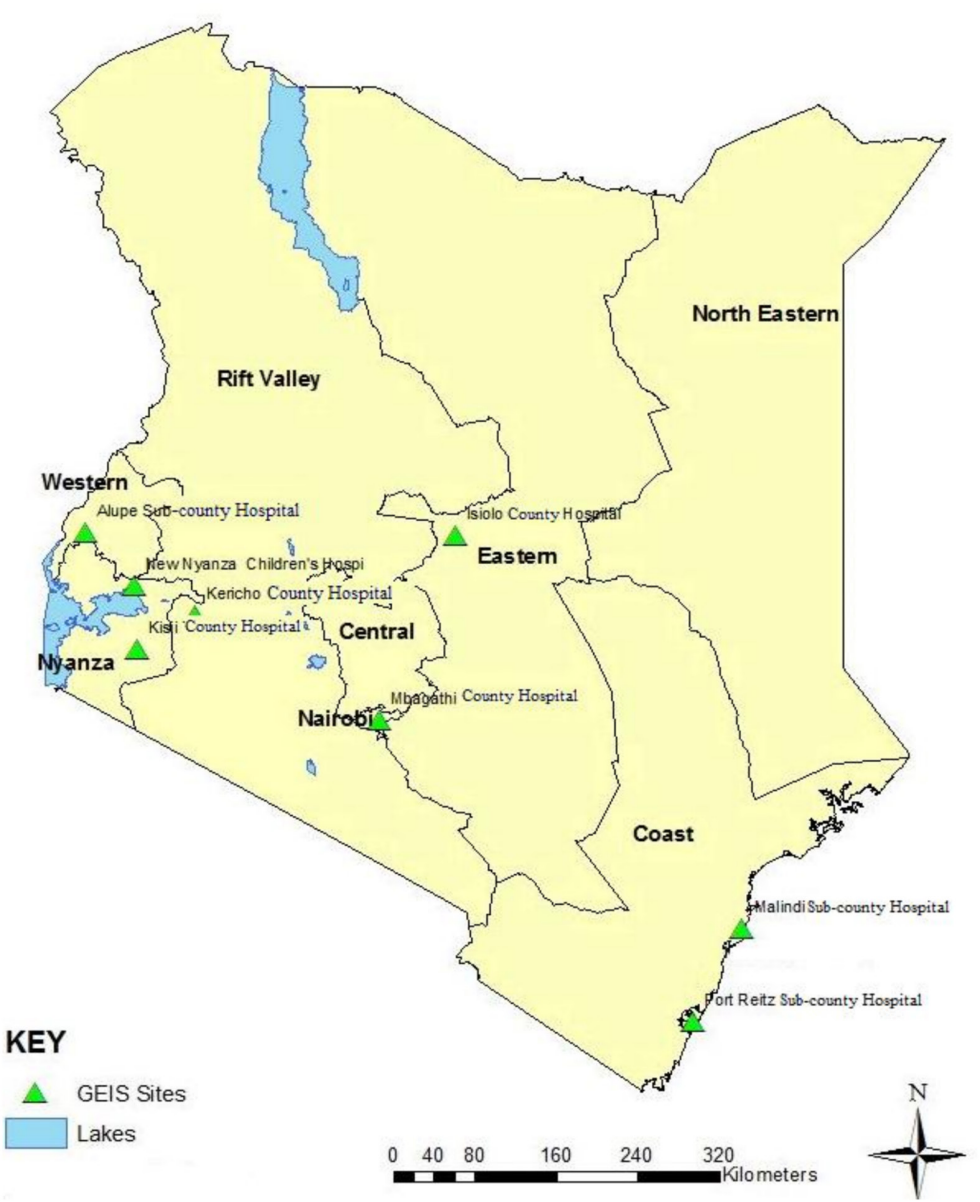
Kenyan map showing the locations of health facilities in the previously known provinces. The five regions examined in this study comprise of one or two public hospitals. The Port Reitz and Malindi subcounty hospitals serve the Coast region, while the Mbagathi county hospital serves the Nairobi region and the Isiolo county hospital represents the Eastern region. The Highlands region encompasses the Kericho county hospital in the middle belt of the rift valley and the Kisii county hospital in the southeast of the larger Nyanza region. The Western region is represented by the Alupe subcounty hospital and the New Nyanza Children's Hospital, which is located in the northeast of Nyanza province.

This study was carried out within the department of emerging infectious diseases' (DEID) surveillance network of the US Army Medical Research Directorate–Kenya. The network included eight outpatient hospitals spread across the country. The study used 2300 samples from the Kenyan Influenza Surveillance Protocol, which were randomly selected from an estimated 11,000 samples collected between March 2007 and February 2010 [20]. The surveillance network comprised of the following public hospitals: Port Reitz and Malindi (Coast region), Mbagathi (Nairobi region), Isiolo (Eastern region), Kisii and Kericho (Highlands region), and Alupe and New Nyanza Children's hospitals (Western region) (Figure 1) [20].

### 2.2 Ethics Statement

This study was approved by the Kenya Medical Research Institute's Scientific and Ethical Review Unit (SERU) (SSC 2051) and the Walter Reed Army Institute of Research's Institutional Review Board (IRB) (WRAIR 1267 subprotocol 1).

### 2.3 RNA Extraction, Screening, Amplification, and Nucleotide Sequencing

A previously published approach was utilized to screen for HRSV using modified multiplex real‐time reverse transcription polymerase chain reaction (MPX RT‐PCR). The assay used HRSV group‐specific Taqman primers (HRSV‐A and HRSV‐B) and a primer specific to adenoviruses [21, 22].

The cDNA was obtained from 5 μL of the MPX RT‐PCR positive RNAs in a 25‐μL reaction assay utilizing the QIAGEN one‐step Reverse Transcriptase Polymerase Chain Reaction (RT‐PCT) kit. To amplify the larger PCR fragment containing a portion of the second domain of the G‐gene, the third domain of the G‐gene, and a portion of the F‐gene, the reaction used two flanking primers consisting of a forward primer, AG20 (5′‐GGGGCAAATGCAAACATGTCC‐3′), and reverse primer, F164 (5′‐GTTATGACACTGGTATACCAACC‐3′). The reaction involved the following steps: 1 cycle of 30 min at 50°C, 1 cycle of 15 min at 95°C, 40 cycles of (30 s at 94°C followed by 54°C for 30 s and 1 min at 72°C) and 10 min at 72°C.

Thereafter, 2 μL of the One‐Step RT‐PCR product was utilized in a nested PCR reaction to amplify the third domain of the G‐gene containing the first hypervariable region, conserved region, and the second hypervariable region, using Taqman PCR master mix kit (Qiagen). The reaction utilized a forward primer, BG10 (5′‐GCAATGATAATCTCAACCTC‐3′), and F1 reverse primer (5′‐CAACTCCATTGTTATTTGCC‐3′) [21].

The amplicons were sequenced utilizing the Sanger ABI dideoxy chain termination technology. Four primers were utilized; two of the primers used in the nested PCR (BG10 and F1) and two group‐specific primers, G533F (5′‐TGTAGTATATGTGGCAACAA‐3′) and G533R (5′‐TTGTTGCCACATATACTACA‐3′) [21]. The resulting nucleotide sequences were edited, assembled, and aligned using multiple tools including DNABaser version 3.2 [23] for sequence assembly, BioEdit program version 7.7 [24] and Muscle v3.8.31 software [25] for multiple sequence alignment.

### 2.4 Phylogenetic and Evolution Analysis

These analyses utilized the complete portion of the G‐gene sequence recovered, with length varying from 534 to 594 base pairs depending on the virus. We aligned 73 local sequences with 31 global sequences of known genotypes retrieved from GenBank for up to 2017. The criteria used to curate these sequences include (i) highest percentage nucleotide identity and (ii) length that is equals to the local sequences.

The phylogenetic trees were inferred using MrBayes version 3.2 utilizing the Metropolis‐coupled Markov chain Monte Carlo (MC3) algorithm [26, 27]. Further selection was conducted to exclude multiple global sequences in a single cluster and those clusters devoid of local sequences in the final presentation of the phylogenetic tree.

MEGA 11 was utilized to compute the genetic diversity within the population, evaluate the variability of the virus, and to identify the best fit model for subsequent analysis. Models with the lowest Bayesian information criterion (BIC) scores were considered for evolutionary analysis [28].

The evolutionary rate was estimated using the Bayesian Markov chain Monte Carlo (MCMC)–based approach implemented in BEAST v1.8.2 [29]. The evolutionary analysis assumed an uncorrelated lognormal relaxed molecular clock model and implemented the Hasegawa‐Kishino‐Yano and gamma (G) plus invariant (I) nucleotide (HKY + G + I) substitution model. BEAST run convergence confirmation (ESS > 200) and analysis of parameter estimates was accomplished using Tracer v1.6 [30]. The BEAST trees were summarized using TreeAnnotator v1.8.2, and the maximum clade credibility (MCC) tree was visualized using Figtree v1.4.3 software [31].

### 2.5 Selection Pressure and Recombination Analysis

Selection pressure was estimated using four methods implemented in the web based DATAMONKEY application: single likelihood ancestor counting (SLAC), fixed effects likelihood (FEL), fixed unconstrained Bayesian approximation (FUBAR), and mixed effect model of evolution (MEME) [32, 33]. A *p*‐value of 0.01 (SLAC, FEL, and MEME) and a posterior probability of 0.9 (FUBAR) were applied in determining the positively (dN/dS > 1) and negatively (dN/dS < 1) selected sites, respectively. The genetic algorithm for recombination detection (GARD) implemented within the DATAMONKEY application [34] was utilized to detect potential recombination events and thereafter verified using the RDP, BootScan, 3SEQ, Chimaera, and GENECONV methods implemented the Recombination Detection Program version 4.39 (RDP4) [35].

## 3 Results

### 3.1 Detection, Spatial, and Temporal Distribution of HRSV‐B

**FIGURE 2:**
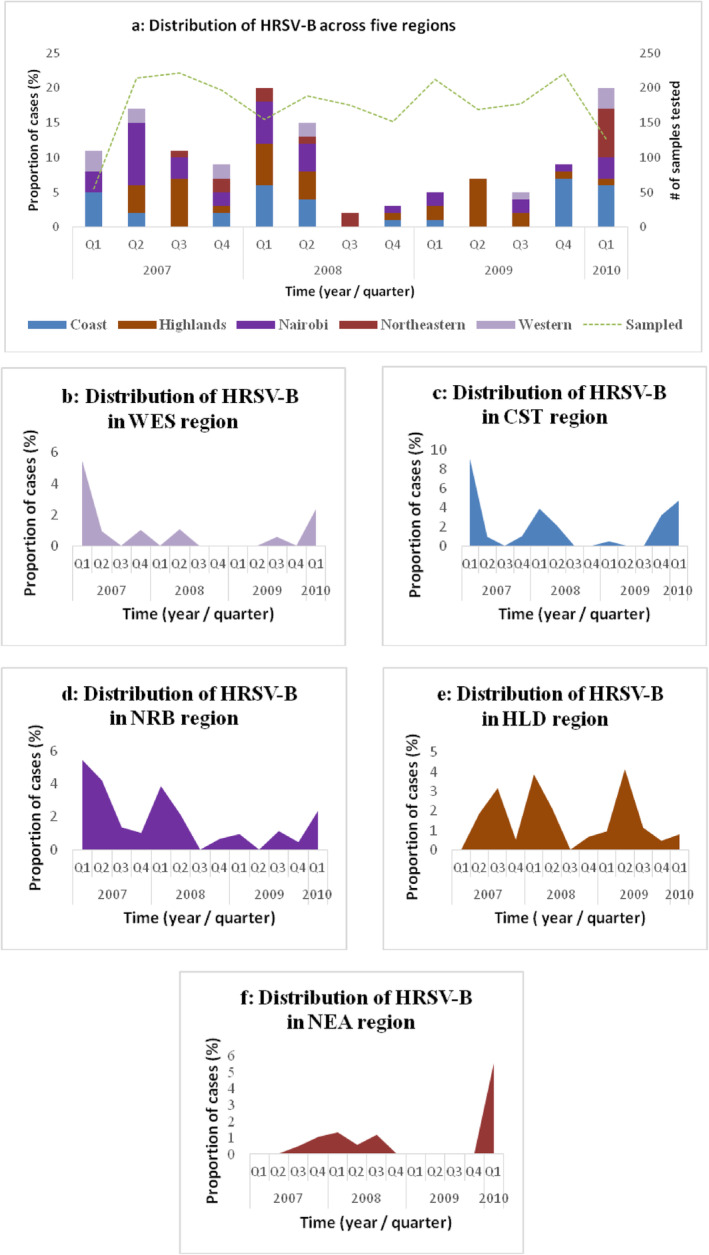
(a–f) Temporal spatial distribution of HRSV‐B in Kenya. (a) Quarterly distribution of HRSV‐B from 2007 to 2010. It is displaying the proportion of cases in the five regions and the total number of samples tested per quarter. The quarters followed the calendar year starting January to December. (b–f) Temporal distribution of HRSV‐B in five regions. (b) Western (WES), (c) Coast (CST), (d) Nairobi (NRB), (e) Highlands (HLD), and (f) Eastern (NEA).

Of the 2300 clinical samples tested, 145 (6.3%) were positive for HRSV‐B. The virus was observed in varying proportions with distinct peaks each year. HRSV‐B cases were detected in all regions during Q2 and Q1 of 2008 and 2010, respectively (Figure 2a).

HRSV‐B cases were highest in the Coast and Nairobi regions during the first quarter with a variation seen in 2009 (Figure 2c,d). The incident peaks varied in highlands region from 2007 to 2009 (Figure 2e). Quarters 1 of 2007 and 2010 exhibited the highest incidence in the Western region (Figure 2b). The lowest incidence was observed in 2009, with no cases reported from the Eastern region (Figure 2f). This analysis revealed an upswing in HRSV‐B activity during the initial quarter of 2010 in four of the regions, except for the Highlands region, where there was a declining trend.

### 3.2 Characterization of HRSV‐B and Genotype Distribution in Kenya, 2007–2010

**FIGURE 3:**
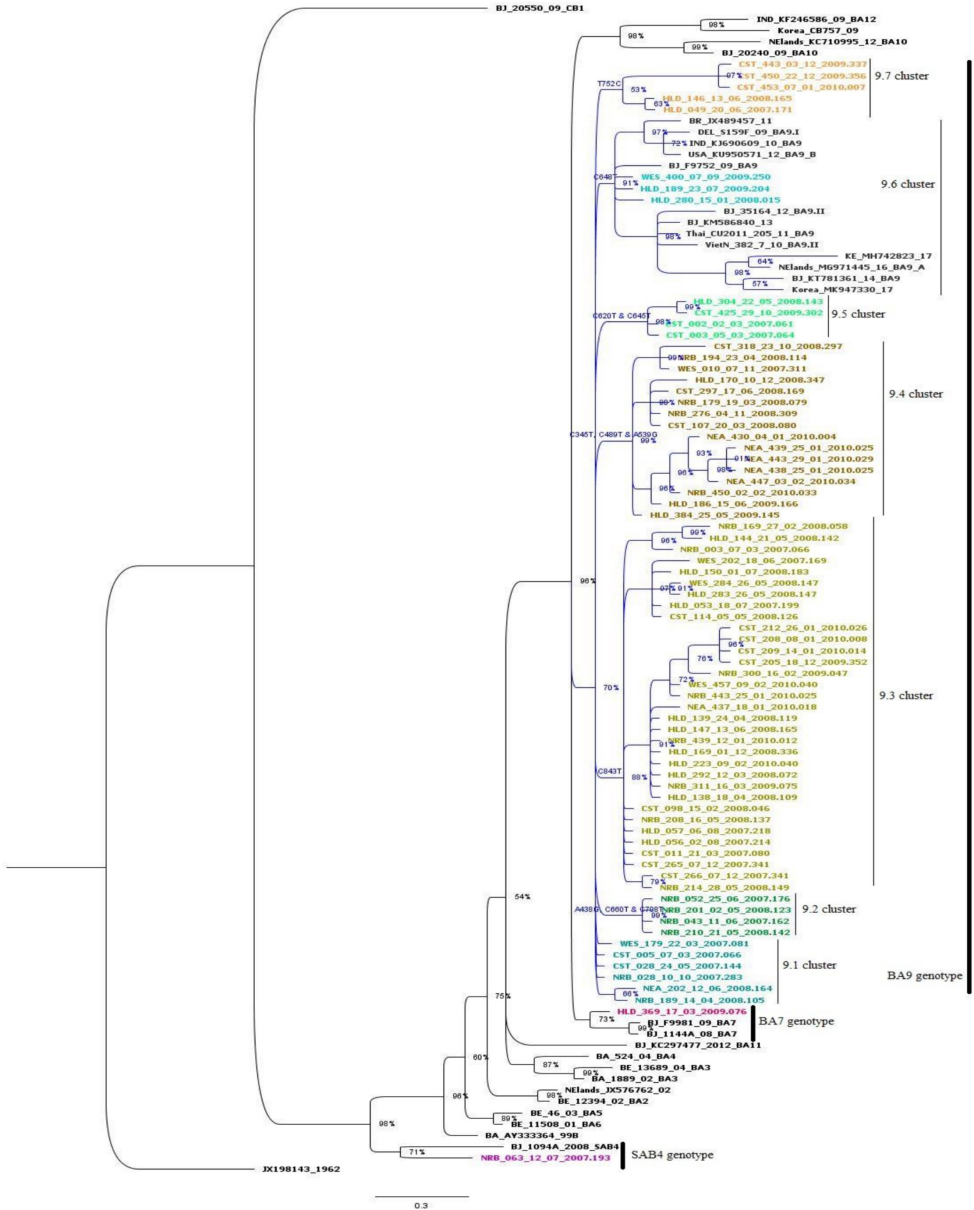
Phylogenetic tree based on the G‐gene segment of the HRSV‐B recovered from the ILI cases across Kenya. It is made up of 104 sequences rooted on the prototype isolated in 1962, accession number JX198143. The local sequences (73) are represented by the colored tips. Each individual cluster or subcluster has a distinct color for the tips. We only assigned genotype names to clusters comprising local sequences. The BA9 genotype comprised of seven clusters labeled as BA9.1–BA9.7. The nucleotide differences among these sequences were annotated based on the prototype sequence and therefore does not display the changes in the duplicated segment among these sequences.

The sequences were deposited in the GenBank (accession numbers PP101687–PP101759). The phylogenetic analysis showed that 71 of these sequences (97.2%) formed the BA9 cluster, followed by two sequences, (2.8%) clustering with the BA7 and SAB4 genotypes each (Figure 3). In the Nairobi region, SAB4 and BA9 genotypes were identified, while BA7 and BA9 genotypes were detected in the Highlands. Conversely, only the BA9 genotype was observed in the Coast, Eastern, and Western regions with the lowest cases seen in the Eastern (10%) and Western (9%) regions.

The phylogenetic tree displays BA9 clade, (clade credibility value of 70%), delineated into seven clusters which we designated as BA9.1–BA9.7. When compared to the prototype (JX198143), these clusters presented distinct characteristics at the nucleotide level and amino acid level. The BA9.1 cluster was made up of four sequences that remained on the BA9 clade as well as two sequences from an adjacent cluster. The 9.1 and 9.2 clusters comprised sequences collected during the first two years of the study, whereas 9.3 cluster included sequences collected throughout the study. This was the most extensive of the BA9 clusters, encompassing 33 sequences, distinguished by a singular nucleotide substitution, C843T.

The sequences in the remaining four clusters were collected over 2–4 years of the study. Markedly, three of the clusters (BA9.4, BA9.5, and BA9.7) showed features marked by one to three nucleotide substitutions with an amino acid change. The BA9.4 cluster exhibited three nucleotide substitutions leading to one amino acid change: A345T, C489T, and A539G (resulting in Q180R). On the other hand, BA9.7 cluster showed a single nucleotide change, T752C, (resulting in V251A) whereas BA9.5 cluster exhibited two nucleotide changes and one amino acid change: C620T (resulting in T207L) and C645T.

Remarkably, only three of the local sequences in the BA9 genotype clustered with global sequences collected between 2009 and 2017, as seen in Figure 3, cluster BA9.6. These sequences showed a single nucleotide mutation (C648T) and were collected in 2008 and 2009.

### 3.3 Genetic Analysis and Amino Acids Deduction

In the realm of genetic analysis and amino acid deduction, the mean distance within the BA subgroup (BA7 and BA9 genotypes shown in Figure 3) extended up 0.008 with an overall distance mean of 0.013. Conversely, the mean distance between the BA9 clusters exhibited a range of 0.005 to 0.021 (Table 1).

**TABLE 1:** The group means between the clusters within the BA9 genotype.

	BA9.7	BA9.5	BA9.4	BA9.3	BA9.2	BA9.6	BA9.1
BA9.7							
BA9.5	0.01816						
BA9.4	0.02102	0.02009					
BA9.3	0.01673	0.01688	0.01779				
BA9.2	0.01905	0.01750	0.01716	0.01500			
BA9.6	0.01603	0.01451	0.01405	0.01202	0.01105		
BA9.1	0.01265	0.01151	0.01103	0.00905	0.00807	0.00458	

*Note:* The analysis utilized MEGA 11 for evolutionary divergence using 71 sequences. The 7 clusters within the BA9 genotype were considered as groups in this analysis.

The Kenyan BA subgroup exhibited amino acid variability in comparison to the BA prototype (AY333364), with variations observed in 30 out of the 196 amino acids. All the local sequences had a 3‐amino acid deletion at positions 157–159 and 4 substitutions at the following positions: Y112H, K218T, L223P, and S247P (Figure 5a,b). These sequences displayed mutations within the region of 20‐amino acid duplication (comprising a 60‐nucleotide duplication), amounting to a total of seven mutations. Among the Kenyan sequences, significant mutations were identified at positions 11 (T to I) and 12 (V to A), observed in 62 (86%) and 69 (95%) of the sequences, respectively (Figure 5b).

### 3.4 Evolution, Selection Pressure, and Recombination Analyses

The mean evolutionary rate for the local HRSV‐B was estimated at 3.08 × 10^−3^ substitutions per site per year (95% high posterior density [HPD], 1.81 × 10^−3^ to 6.48 × 10^−4^) during the study period. We further estimated the mean evolutionary rate for the BA subgroup at 2.78 × 10^−3^ substitutions per site per year (95% high posterior density [HPD], 1.65 × 10^−3^ to 6.77 × 10^−4^).

**FIGURE 4:**
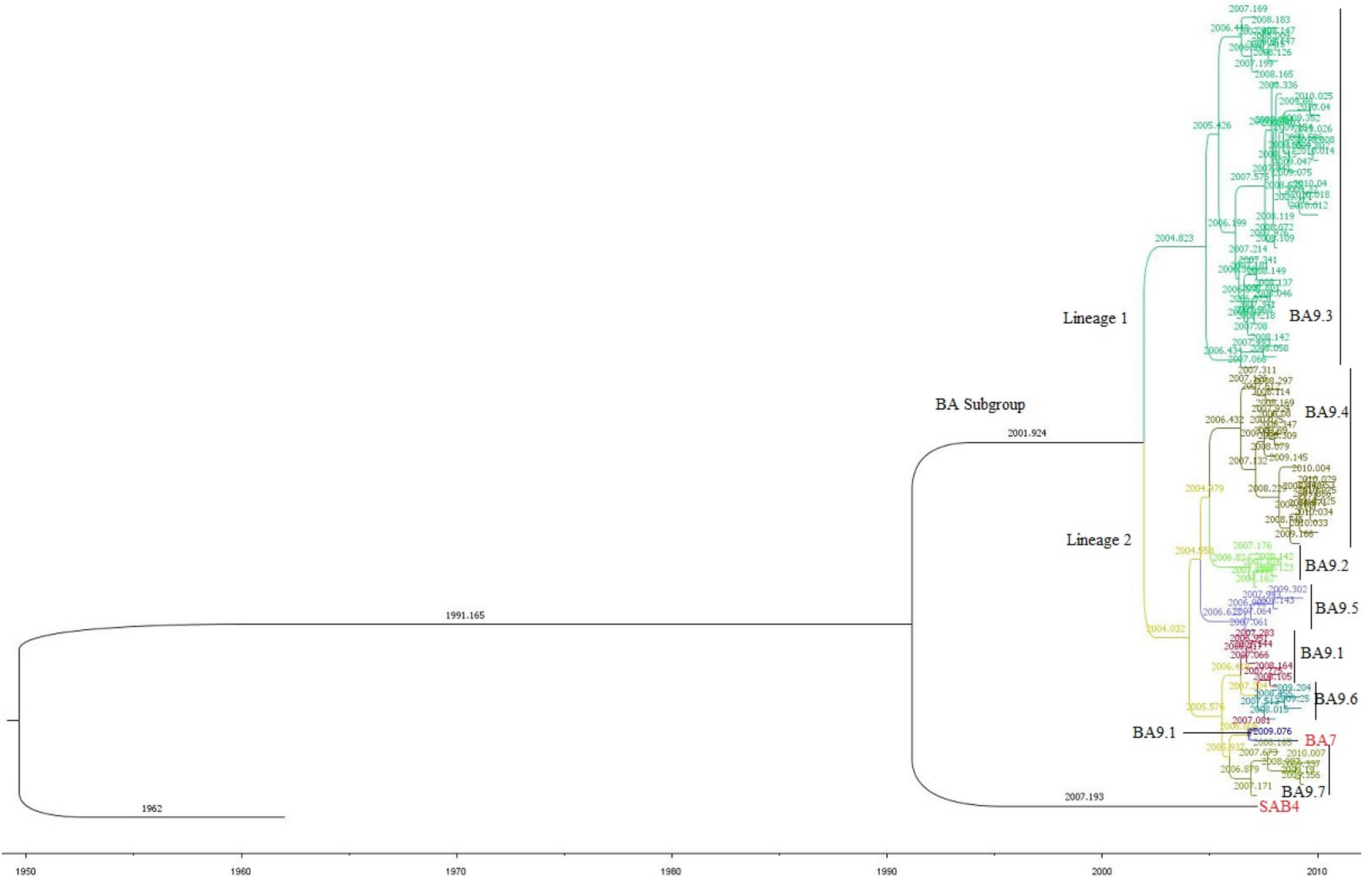
Bayesian Markov chain Monte Carlo (MCMC) tree based on the G‐gene segment of the HRSV‐B recovered from the ILI cases across Kenya. The tree comprised of 74 sequences. It is rooted on the prototype, accession number JX198143. The two major lineages are represented by distinct colored clades. Lineage 1 comprises of 33 sequences found in cluster 9.3 of Figure 2 while lineage 2 comprises of the remaining 38 BA9 sequences and 1 BA7 sequence represented in different shades. The BA9 clusters shown in Figure 2 are labeled in black font while the BA7 and SAB4 genotypes are labeled in a colored font.

The Bayesian MCMC analysis showed that the local SAB4 genotype first emerged around 1990. A major divergence took place around 2001 leading to the emergence of two distinct lineages within the BA subgroup (Figures 4 and S1). In 2004, each of the two lineages diverged further, resulting in two distinct sublineages. Following this split, further diversification occurred, leading to the appearance of the BA7 genotype in 2006 among other sublineages.

**FIGURE 5:**
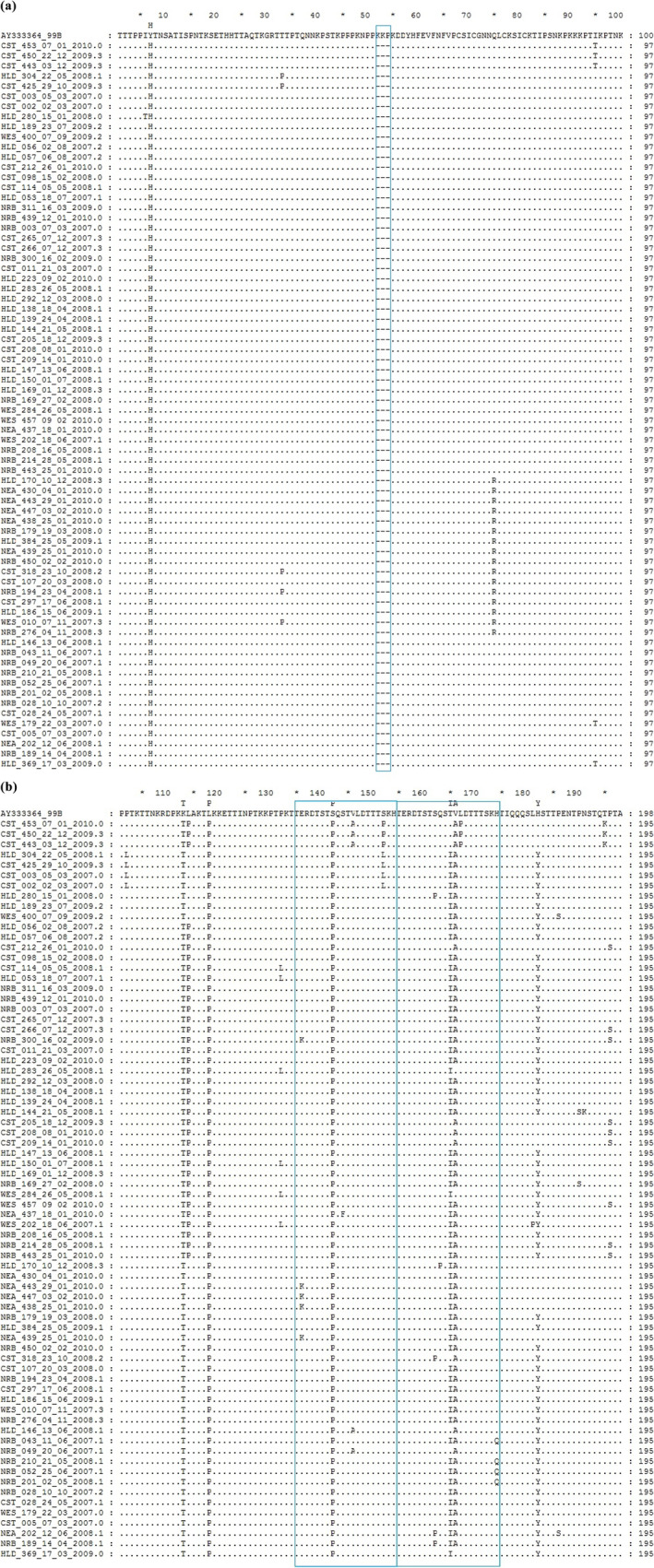
Sequences alignment of a segment of deduced amino acids of a segment of the G‐gene of the HRSV‐B sub‐group BA. The alignment shown is relative to a BA isolate from Argentina, GenBank Accession number AY333364. (a) The alignment covers 100 residues of the first hypervariable region starting from position 107 of the reference strain. The box frame represents a deletion in the local sequences. (b) This alignment covers the second hypervariable region from position 101, which is equivalent to position 208 of the reference strain. The boxes frame covers a region of 40 amino acids in the duplicated region. The box frame to the right is a copy of the 20‐amino acids insertion.

The FEL, MEME, and FUBAR models concurred in pinpointing two specific sites (270 and 287) subjected to episodic and pervasive diversification (Table 2). The SLAC model identified three sites subjected to purifying selection and none under positive selection. In contrast, the FEL model revealed the potential influence of selection on up to 11 codons. The three selection models were further used to analyze sites under selection pressure for the population by year of collection (Table 2). Codon 270 was seen to be under diversification by at least two models in 2008 and 2010. This residue was under both pervasive diversification and episodic diversification in 2008.

**TABLE 2:** The residues of HRSV‐BA subgroup under positive selection across Kenya from 2007 to 2010.

Methods	Positively selected sites per year				Amino acid substitution
	2007	2008	2009	2010	
SLAC	None	None	None	None	NA
FEL	270^a^	270^a^	None	270	T270I
FUBAR	None	270^a^	270, 287^a^	270, 287^a^	T270I, H287Y
MEME	None	270^a^	None	None	T270I

*Note:* The residue position is in reference to a reference strain from Argentina, AY333364. The *p* value of 0.01 was used for SLAC, MEME, and FEL, while the FUBAR model had a posterior probability of 0.9.

Abbreviation: NA: not applicable because there were no positively selected sites during the year under evaluation.

^a^ The codon is located within the duplicated region of the G gene.

The genetic algorithm for recombination detection (GARD) program detected a potential breakpoint while no recombination events were detected by the RDP, BootScan, 3SEQ, Chimaera, and GENECONV methods in the Recombination Detection Program version 4.39.

## 4 Discussion

The current research offers a comprehensive analysis of the spatiotemporal distribution patterns, genetic diversity and molecular evolution of human respiratory syncytial virus B (HRSV‐B) among individuals exhibiting acute respiratory illness with influenza‐like symptoms in Kenya. This investigation is particularly pertinent, as it took place during a pivotal historical period marked by the global spread of a new pandemic influenza variant. The study period aligns with the Influenza A/H1N1/2009 outbreak and precedes the widespread emergence of various HRSV‐B genotypes. Spanning from March 2007 to February 2010, the timeframe captures key developments in the evolution and distribution of HRSV‐B during a period of significant global health concern.

Our data were grouped in quarters following the calendar year due to the low monthly number of HRSV‐B cases. The geographic temporal distribution pattern varied by location; however, certain regions showed comparable patterns. This analysis reveals variable seasonal patterns in the country with three to five HRSV‐B peaks from March 2007 to February 2010. First, in the Nairobi region, the highest HRSV‐B activity was in the first quarter (January–March), with the lowest in the fourth quarter (October–December). Second, the highest activity in the Coast region was in the first quarter with the lowest occurrences seen during the third quarter (July–September). Finally, the highest activity in the Western and Highlands regions demonstrated a dynamic pattern of fluctuation ranging from the first to third quarters. The virus was detected in low proportions in the Eastern region with a notable increase in transmission at the end of the study period.

There was reduced or no HRSV‐B activity in 2009 across the regions. While the reasons are not clear, they may be attributed to (i) a biennial HRSV cycle with significant decline of HRSV‐B during the year [36] and (ii) low HRSV incidences during the high Influenza activity as reported in the Western Kenya [37]. A study utilizing same sample population recorded the highest influenza activity during the third quarter with Influenza/H1N1/2009 recording the highest incidence in the third and fourth quarters within this demographic [38]. This study highlights the molecular epidemiology of HRSV‐B during the Influenza/H1N1/2009 pandemic, offering insights into the ecological dynamics of respiratory viruses. The reduced HRSV‐B activity during that period suggests possible viral interference or host immune interactions.

Similar patterns emerged during the COVID‐19 pandemic, where SARS‐CoV‐2 significantly altered RSV transmission, leading to delayed or off‐season outbreaks due to non‐pharmaceutical interventions (NPIs), shifts in population immunity, and viral interactions [39, 40, 41]. While our study predates SARS‐CoV‐2 by a decade, the parallels underscore how novel respiratory pathogens can disrupt endemic virus epidemiology [42]. These findings emphasize the need to investigate interactions between emerging and seasonal viruses to better inform surveillance, vaccination, and outbreak preparedness strategies.

HRSV‐B with 60 nucleotide duplication in the second hypervariable region of G gene was first reported in 1999 [43]. This strain, BA subgroup, was reported to have gradually replaced the existing genotypes within the decade in most of the continents [44]. The current study has identified one sample belonging to the SAB4 genotype, a non‐BA group collected in 2007. SAB4 genotype has been sporadically detected elsewhere in the past decade [15, 17, 45]. These findings indicate that the BA subgroup had not fully supplanted the pre‐existing genotypes. The HRSV‐B, BA subgroup has evolved to over 12 genotypes (BA1–BA12) to date [10, 19, 46]. During the last decade, the BA9 genotype has been reported to be gradually increasing its global dominance [17, 46, 47]. It was the predominant genotype during the period of this study (2007–2010) accounting for over 95% of the sequences, consistent with other studies worldwide [48, 49]. It is worth noting that it belongs to the newly proposed GB5.0.5a genotype [9].

We further evaluated the BA9 genotype using a broader definition approach of variants, which are distinct strains with specific mutations in comparison to the prototype strain. We therefore recognize the previously identified 9.1–9.7 BA9 clusters in Figure 3 as variants. These variants differed substantially, forming seven variants (BA9.1–BA9.7) with a genetic distance (P‐distance) of < 0.3. Notably, only the BA9.6 variants clustered with sequences from elsewhere in four subvariants: (i) BA9.A from Netherlands, (ii) BA9.B and BA9.I from the United States and India respectively, (iii) BA9.II from China and Vietnam, and (iv) local variant alongside with a variant from China. The current classification criterion is based on monophyletic clusters with > 70% bootstrap value and < 0.07 inter genotypic distances of based on full genome analysis [9, 35, 46, 50].

The genetic changes in a variant can alter viral features, potentially increasing prevalence in certain populations thus classifying high risk variants as variant of concern [8, 12, 51]. While our study did not evaluate the viral characteristics of the local variants, we utilized the Bayesian MCMC analysis to illustrate the ancestor‐offspring relationship (lineages) among the BA subgroup (proposed GB5.0.1 genotype) [9]. The BA9.3 variant represented the earliest divergence of the Bayesian MCMC tree. It appeared in 2005 thus representing the first lineage among the local BA subgroup (shown in Figure 4). The remaining BA9 variants (9.1, 9.2, 9.4, 9.5, 9.6, and 9.7) and the BA7 genotype made up the second lineage. The BA9.2 and BA9.5 variants appeared to be the oldest in the second lineage, followed by the BA7 genotype and the youngest being the BA9.4 variant.

Viral evolution rates vary due to regional factors and population immunity, as evidenced by the disparity in HRSV‐B evolution rates across the globe over time [45, 52]. The local BA9 genotype had a slower mean evolution rate of 2.56 × 10^−3^ substitutions/site/year in comparison with those from elsewhere. For example, during the 2006–2016 time frame, China and India reported a mean evolutionary rate of 4.93 × 10^−3^ and 4.58 × 10^−3^ substitutions/site/year, respectively [14, 17].

A number of amino acid variations were observed across the entire segment in comparison with both the prototype sequence (JX198143) and a 1999 BA sequence (AY333364). A mutation at position 180 (Q180R) was observed within the local BA9.4 variant. This mutation was also reported sporadically in other areas [47]. The local BA sequences lacked some of the mutations exhibited by the BA reference strain in positions 112 (H‐Y), 218 (T‐K), and 223 (P‐L), hence similar to the prototype. They further exhibited parallel mutation in position 247 (S247P), which was reported elsewhere [17, 49].

We further observed T270I, V271A, and H287Y substitutions in over 75% of the local sequences, which was consistent with other reports across the globe [17, 49, 50, 53]. Worth noting, two of the above mutations were found in the 20 amino acid duplication segment (which starts from 260 up to 279). The V271A and H287Y mutation were also present in the local BA7 sequence and thus not restricted to the BA9 sequences [54].

While the G gene is known to be the most variable gene in the HRSV genome, the current study revealed only two sites under diversifying selection using FEL, FUBAR, and MEME models. While none of the sites were under diversification in 2007, site 270 (threonine to isoleucine) was seen to be under both pervasive and episodic diversification in 2008 by the three models. A second site, 287 (histidine to tyrosine), was identified to be under diversification in 2009 and 2010 using the MEME model expressing episodic diversification. This site was reported elsewhere to be undergoing diversification during the same time frame [53]. Diversifying selection displays a survival advantage under the selective constraints that confront the viral population. Additionally, there was no evidence of recombination in this population. It is worthwhile noting that recombination events are known to be a rare in HRSV [12, 55].

The limitation of the study was mainly on the low numbers of cases and also the sampling period, as samples collected in 2010 were limited to the first 2 months as indicated in the methods and therefore lacking a holistic picture of the HRSV activity at the peak of the Influenza A/H1N1/2009 pandemic.

## 5 Conclusions

The genetic profiling of HRSV‐B reveals the existence of a non‐BA subgroup at a time when it was almost extinct, alongside dominance of the BA9 genotype. It sheds more light on the local strain's significant genetic diversity. It emphasizes the uniqueness of the local BA9 genotype, as well as variants that are uniquely Kenyan which calls into question the possibility of unreported genotypes in the country.

## Author Contributions

**Julia Wangui:** conceptualization, investigation, writing – original draft, methodology, validation, visualization, writing – review and editing, formal analysis, project administration, data curation. **George Gachara:** formal analysis, data curation, software, validation, visualization, writing – review and editing, methodology. **Victor Mobegi:** writing – review and editing, data curation, formal analysis. **Charles Agoti:** conceptualization, methodology, data curation. **James Otieno:** validation, data curation, writing – review and editing. **Silvanos Opanda:** writing – review and editing, data curation, formal analysis, methodology. **Benjamin Opot:** data curation, formal analysis, software. **Joseph N. Ngeranwa:** supervision, conceptualization, writing – review and editing. **Regina Njeru:** methodology, validation. **Wallace Bulimo:** conceptualization, investigation, funding acquisition, writing – review and editing, supervision, resources, data curation, formal analysis.

## Disclosure

The views or insertions expressed herein are private views of the authors and are not to be construed to represent those of the US Department of Defense or Army.

## Conflicts of Interest

The authors declare no conflicts of interest.

### Peer Review

The peer review history for this article is available at https://www.webofscience.com/api/gateway/wos/peer‐review/10.1111/irv.70082.

## Supporting information


**FIGURE S1** Bayesian Markov Chain Monte Carlo (MCMC) tree based on the G‐gene segment of the HRSV‐B recovered from the ILI cases across Kenya. The tree comprised of 74 sequences. It is rooted on the prototype, accession number JX198143. The two major lineages are represented by distinct colored clades. Lineage 1 comprises of 33 sequences found in cluster 9.3 of Figure 2 while lineage 2 comprises of the remaining 38 BA9 sequences and 1 BA7 sequence represented in different shades. The BA9 clusters shown in Figure 2 are labeled in black font while the BA7 and SAB4 genotypes are labeled in a colored font. The tips display the sequence names.

## Data Availability

The data presented is available in the National Center for Biotechnology Information using the following GenBank accession numbers: PP101687 ‐ PP101759.
