# Supplementary figures and images for: Molecular Analysis of Human Respiratory Syncytial Virus Group B Strains Isolated in Kenya Before and During the Emergence of Pandemic Influenza A/H1N1
Source: Influenza Other Respir Viruses. 2025 Feb 20;19(2):e70082. doi: 10.1111/irv.70082 (PMC11842092; doi:10.1111/irv.70082)

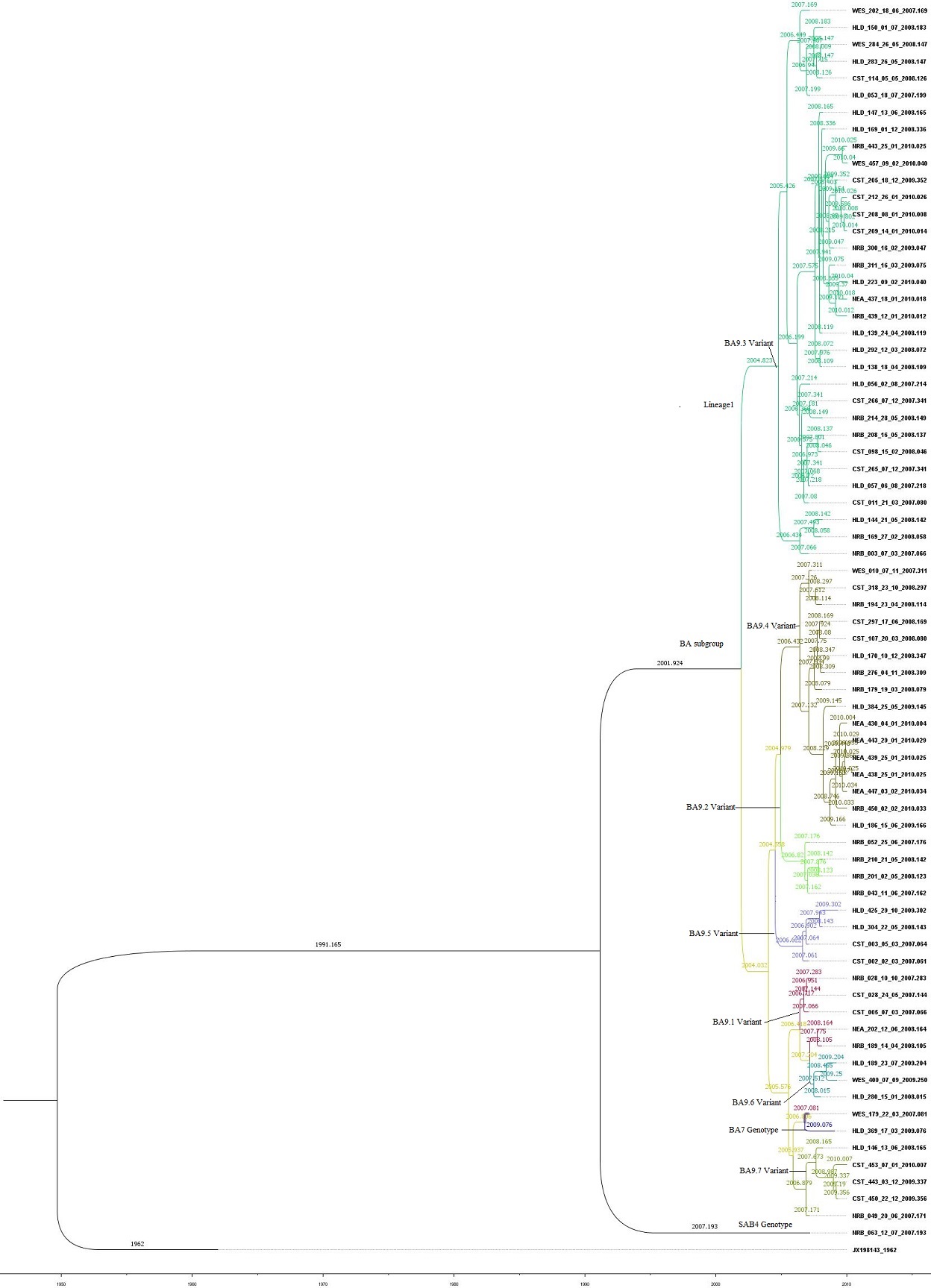

Supplement: Supplementary file 1 — FIGURE S1 Bayesian Markov Chain Monte Carlo (MCMC) tree based on the G‐gene segment of the HRSV‐B recovered from the ILI cases across Kenya. The tree comprised of 74 sequences. It is rooted on the prototype, accession number JX198143. The two major lineages are represented by distinct colored clades. Lineage 1 comprises of 33 sequences found in cluster 9.3 of Figure 2 while lineage 2 comprises of the remaining 38 BA9 sequences and 1 BA7 sequence represented in different shades. The BA9 clusters shown in Figure 2 are labeled in black font while the BA7 and SAB4 genotypes are labeled in a colored font. The tips display the sequence names. [file IRV-19-e70082-s001.jpg]
